# Climate Impacts of Deforestation/Land-Use Changes in Central South America in the PRECIS Regional Climate Model: Mean Precipitation and Temperature Response to Present and Future Deforestation Scenarios

**DOI:** 10.1100/2012/972672

**Published:** 2012-05-03

**Authors:** Pablo O. Canziani, Gerardo Carbajal Benitez

**Affiliations:** ^1^Equipo Interdisciplinario para el Estudio de Procesos Atmosféricos en el Cambio Global, PEPACG, Facultad de Ciencias Fisicomatemáticas e Ingeniería, Pontificia Universidad Católica Argentina, C1107AFF Ciudad Autónoma de Buenos Aires, Argentina; ^2^Consejo Nacional de Investigaciones Científicas y Técnicas, CONICET, C1033AAJ Ciudad Autónoma de Buenos Aires, Argentina; ^3^Servicio Meteorológico Nacional (SMN), C1002 Ciudad Autónoma de Buenos Aires, Argentina

## Abstract

Deforestation/land-use changes are major drivers of regional climate change in central South America, impacting upon Amazonia and Gran Chaco ecoregions. Most experimental and modeling studies have focused on the resulting perturbations within Amazonia. Using the Regional Climate Model PRECIS, driven by ERA-40 reanalysis and ECHAM4 Baseline model for the period 1961–2000 (40-year runs), potential effects of deforestation/land-use changes in these and other neighboring ecoregions are evaluated. Current 2002 and estimated 2030 land-use scenarios are used to assess PRECIS's response during 1960–2000. ERA-40 and ECHAM4 Baseline driven runs yield similar results. Precipitation changes for 2002 and 2030 land-use scenarios, while significant within deforested areas, do not result in significant regional changes. For temperature significant changes are found within deforested areas and beyond, with major temperature enhancements during winter and spring. Given the current climate, primary effects of deforestation/land-use changes remain mostly confined to the tropical latitudes of Gran Chaco, and Amazonia.

## 1. Introduction

Climate change is primarily associated with greenhouse gas (GHGs) emissions. Nevertheless climate and surface vegetation have bidirectional interactions on different temporal and spatial scales. Hence changes in vegetation distribution and structure can influence climate. Land-use changes are among the primary forcings of climate change, both at regional and global scales [1, 2], among others. Similarly, climate changes can impact the current global vegetation distribution and will further modify it in the future [3].

Land-use change in central and southern South America, primarily in the Amazon and Rio de la Plata Basins, is a common practice, due to expanding agricultural activities resulting from the growing global demand for agricultural commodities, soybean, beef, and raw materials for biofuels, for example, sugar cane, corn, jatropha, and soy beans [4]. Rising commodity prices and production growth of first-generation biofuels have led to enhanced deforestation and savannah losses in Mercosur countries (Brazil, Paraguay and Argentina), as well as in Bolivia. MODIS fire observations in the region [5, 6] highlight the magnitude of this regional process. International commodity demand trends impact upon the region's land-use through linkages that Nepstad et al. [7] call “economic teleconnections.”

These two river basins encompass two major ecoregions, that is, Amazonia and the Gran Chaco, but the adjacent Selva Paranaense/Mata Atlantica (also known as the Atlantic Rainforest) and the Cerrado ecoregions are also undergoing similar processes. The Gran Chaco ecosystem includes both the Chaco Húmedo, with forests, savannas, and wetlands, and Chaco Seco, with dry, sparser forests, brush land, and grasslands. In all these ecoregions large areas of tropical and subtropical rainforest, dry forest/brush land and savannas, have been replaced by pasture, sugar-cane, and more recently by soybean cropland. Such changes are not limited to Amazonia. In the Gran Chaco ecoregion extensive land-use changes have taken place during the last 10–15 years, with high economic profit, albeit with heavy, significant environmental and social consequences.

Such processes have significant environmental and climate impacts. Forests are fundamental for the basins' hydrological cycle in a number of ways. Trees canopies limit soil erosion severe rainfall can cause together with their large root systems, furthermore regulating rainfall drainage towards streams and rivers. Moderate, localized deforestation may locally enhance convection and rainfall due to the resulting local/regional temperature and evaporation changes. Using the CPTEC-INPE global atmospheric model, Sampaio et al. [8] demonstrated that land-use changes for pasture and soybean cropland expansion can lead to increases in near-surface air temperature due to increased surface sensible heat flux and decreases in evapotranspiration and precipitation, changes which mainly occur during the dry season (winter). Malhi et al. ([9] and references therein) observed that land-use changes may reduce cloudiness, increasing insolation, and enhance land surface reflectance. Such activities also lead to changes in atmospheric aerosol loading, from an originally extremely clean atmosphere to a smoky/dusty one, particularly during the dry season: such particulates can in turn further modify rainfall patterns. Savannas are just as important for the hydrological cycle. Both forests and savannas are major CO_2_ sinks, and human-induced modifications can reduce this highly relevant environmental service.

In order to model and assess regional climatic consequences of land-use changes, given their local/regional spatial characteristics, it is necessary to work with a much higher-resolution than that provided by global circulation models (GCMs). Local/regional climate issues are better addressed, in the current state-of-the-art modeling, with higher resolution (~20–50 km) Regional Climate Models (RMCs) such as RegCM (ICTP, Italy) or PRECIS (Hadley Centre, UK), to name a few.

For South America, most local/regional studies have addressed the Amazonian deforestation/land-use change processes, in particular through a number of major field studies such as the Anglo-Brazilian Amazonian Climate Observation Study, ABRACOS [10], and the Large-Scale Biosphere-Atmosphere Experiment in Amazonia, LBA [11]. A number of recent studies including [12–14] have used RCMs to evaluate various aspects of climate processes occurring within Amazonia. More recently, Medvigy et al. [15] carried out a study with the sophisticated variable resolution OLAM general circulation climate model, running it in mesoscale resolution over central South America. Note that OLAM is not an RCM since it is a full-scale GCM which is run in mesoscale resolution over a selected area.

**Figure 1:**
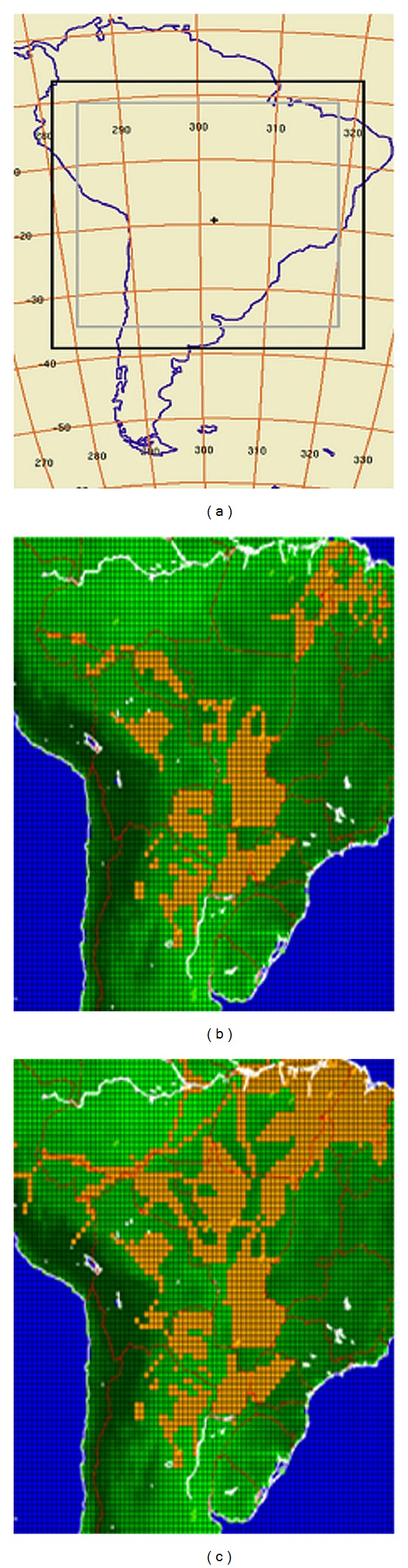
Model domain over central South America. Deforestation (land-use/change scenarios selected for the study. The areas shown in orange represent the land-use/change from forests/savannas to crop fields. The map (b) shows observed land-use changes up to 2002 (MAP1), while the map (c) shows a hypothetical vegetation map for 2030 (MAP2) based on land-use change projections.

However, few have focused in extending the analysis of deforestation/land-use changes in the continent to include similar processes occurring within the Gran Chaco and the Selva Paranaense/Mata Atlantica regions. Thus, the aim of this work is to specifically assess the response to deforestation/land-use change taking place both in Amazonia, to the south of the Amazon River, and in the ecosystems within the Rio de la Plata Basin in RCM runs and, as a corollary, assess the potential consequences of such processes to Central South America's climate. RCM PRECIS, developed at the Hadley Centre, UK, is used here, driven by ERA-40 reanalysis and AGCM ECHAM4 model outputs. This first study specifically explores PRECIS's mean climate response in 40-year runs to three deforestation/land-use change scenarios (Figure 1) during the baseline period 1961–2000. The scenarios used are the following: (i) the original vegetation map provided for in PRECIS (CONTROL), with land-uses and land covers corresponding to 1978, (ii) the vegetation/land-use map updated with observations to 2002 (MAP1), following Eva et al. [16], and (iii) a hypothetical vegetation/land-use map proposed by Nepstad et al. [7], depicting the potential state of Amazonia in 2030 (MAP2). The consequences of such vegetation and land-use changes, resulting from these scenarios in PRECIS simulations for the 40-year baseline period, are discussed. Such an analysis can provide insights into PRECIS response to deforestation/land-use changes in the region and, after validation, a basis for the analysis and discussion of their potential impacts in central South America's regional climate, as given by the physical mechanisms included in the RCM.

After the validation of PRECIS circulation with ERA-40 and ECHAM4, the study analyses the mean seasonal surface temperature and precipitation changes resulting from the three deforestation/land-use scenarios, comparing the results with other model results and observations.

## 2. Model Description and Methodology

Providing REgional Climate for Impact Studies (PRECIS, http://www.metoffice.gov.uk/precis/intro), developed at UKs Hadley Centre, is a dynamical downscaling, high-resolution climate model, for limited area studies. It can be run with a 0.44° (*∼*50 Km) horizontal resolution, optionally 0.22° (*∼*25 Km), in 19 vertical levels in the atmosphere. It uses hybrid vertical coordinates, running in 5-minute time steps. Model runs can be driven by boundary conditions for the past climate as well as for future climate scenarios. PRECIS can be driven by boundary conditions obtained from ECMWF ERA-40 over the period 1957–2001, as well as ERA15 and NCAR R2 reanalysis, and a number of model scenarios (ECHAM4, ECHAM5, HadAM3P, and a seventeen-member ensemble of perturbed GCMs from the HadCM3 QUMP project), spanning the period 1960–2000 and either the complete XXIst century or the reference period 2070–2100, depending on the AGCM driver selected. Note that in dynamical downscaling models such as PRECIS the model itself carries out the calculations corresponding to the physical processes involved, driven by the lower resolution boundary conditions: the RCM generates new outputs, based on the physics included in the model.

### 2.1. Domain Selection and Land-Use Change Scenarios

Georgi and Mearns [17], and recently Alves and Marengo [18], from now on AM10, emphasize that the choice of an RCM domain must be such that it is both large enough for an RCM to develop its own internal regional scale circulations, but not so large that the mean climate reproduced in the RCM simulation deviates significantly from GCM results in the domain's central area. The domain must also be chosen so that known atmospheric processes contributing to the region's climate within the domain and/or along the boundaries, for example, water vapor flux, are included in model runs.

The domain was chosen, taking into account the above caveats, to include the areas where major land-use changes are taking place in central South America, in Brazil, Paraguay, Bolivia, and Argentina. The domain extends from the Equator to 35°S, between 40°W and 79°W, approximately. The horizontal resolution used is 0.44° × 0.44°, given the comparatively smooth topographical as well as extended land-cover features spanning most of the domain, with grid points *n*_*x*_ = *n*_*y*_ = 95 and the perimeter buffer area, as depicted in the Figure 1(a). The ECHAM4 Baseline 1960–2000 (from now on EC4) and ERA-40 reanalysis (from now on E40) were chosen to carry out the RCM runs spanning in all cases the 1961–2000 40-year period.

In order to assess PRECIS's response to land-use changes the original vegetation map, provided by the Hadley Centre for the year 1978, was modified pixel by pixel to obtain the 2002 and 2030 land-use scenarios: pixels where changes have already taken place or are expected to take place were located and changed from tropical forest to agricultural land according to the information available in [7, 16], respectively, using appropriate land-cover definitions available in PRECIS. The results for these three land-use scenarios are referred to as CONTROL, MAP1 (Figure 1(b)) and MAP2 (Figure 1(c)), followed by the driver tag, that is, EC4 or E40.

The PRECIS baseline runs extended from December 1959 through 2001. The first year of each simulation was excluded in order to avoid spin-up problems. PRECIS outputs used in this study are winds at 850 hPa and 200 hPa, surface temperature T, and precipitation seasonal mean fields.

### 2.2. Model Validation for the Selected Domain and Analysis

In order to assess the performance of PRECIS over the domain under study, the RCM was run with the boundary conditions given by ECMWF ERA-40 reanalysis for the period 1961–2000, E40. The ERA-40 data products available have a 2.5° × 2.5° resolution which is enhanced by the model to 0.44° × 0.44°. The CONTROL-E40 outputs for circulation and dynamics fields are compared with the ECHAM4 Baseline-driven model, CONTROL-EC4, output over the same period.

Validation is carried out for seasonal circulation, temperature and precipitation fields and results compared with AM10. AM10 has validated the GCM HadAM3P-driven PRECIS over a somewhat more extended domain. Temperature and precipitation fields provided by the *Climate Research Unit* (CRU) were used for this validation. The CRU TS2.1 dataset has a 0.5° × 0.5° resolution, so it was regridded by linear interpolation to the 0.44° × 0.44° model output resolution. The difference between CRU and model outputs, or bias was calculated at each grid point for temperature and precipitation.

Mean seasonal model output differences between 2002 MAP1 and 2030 MAP2 runs with respect to the CONTROL run were tested with standard statistical tools to determine when detected differences were statistically significant. These were tested, under the assumption that the multiannual seasonal means have a normal distribution for the variables under study, with either two-tailed Student's *t*-test or single-tailed Student's *t*-test, the latter when the observed differences would be mostly of the same sign, this being the case of temperature.

## 3. Results

### 3.1. Domain Circulation Validation

**Figure 2:**
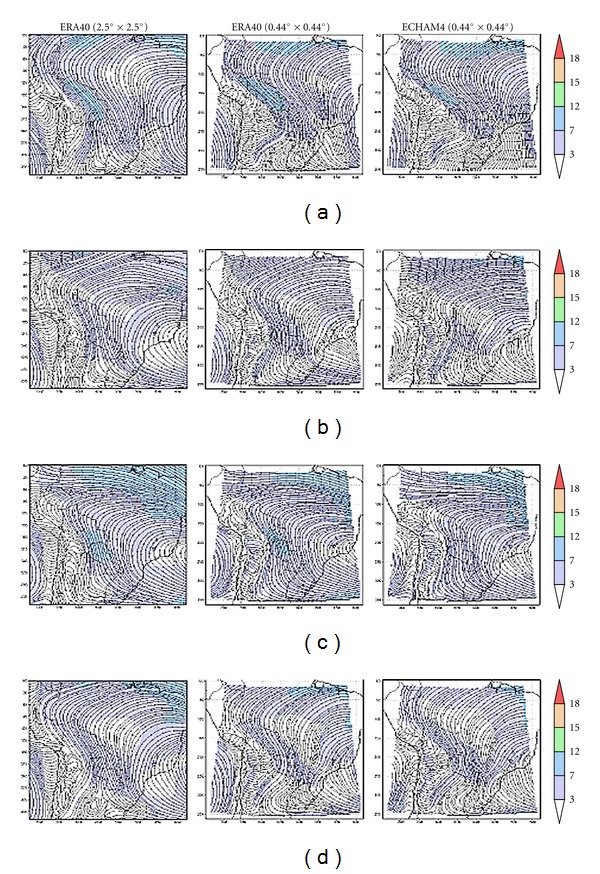
Isolines and wind intensity for 40 years (1961–2000) mean wind field (m/s) at 850 hPa for (a) summer (DJF), (b) autumn (MAM), (c) winter (JJA), and (d) spring (SON). Original ERA-40 2.5° × 2.5° data (left column) and PRECIS outputs for runs driven by ERA-40 C (center column) and ECHAM4 (right column). Wind intensity is shaded.

**Figure 3:**
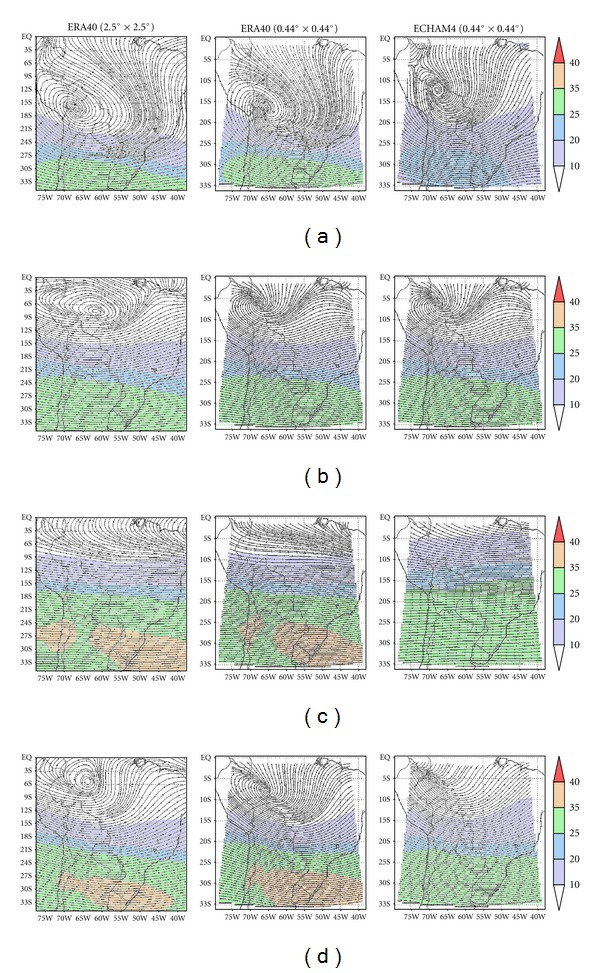
Same as Figure 2 but for upper level winds at 200 hPa.

Inspection of the circulation over the domain, as given by CONTROL-EC4 and CONTROL-E40 runs, was carried out as in AM10, for 850 hPa (low level, Figure 2) and 200 hPa (upper level, Figure 3) mean seasonal wind fields for the period 1961–2000.

During Austral summer (DJF), an important feature of the 850 hPa circulation (Figure 2(a)) over central South America is an easterly/southeasterly trade wind flow in the vicinity of the equator which rotates towards a northerly/northwesterly flow along the eastern slopes of the Andes mountain range and the domain's center and southern sectors. This flow is responsible for the advection of oceanic water vapor into the region, as far south as the Humid Pampas in Argentina and Uruguay, in the domain's south. In this area, the occurrence of the meridional low level jet events, also called Chaco Jet, with a 17% occurrence rate during summer days [19, 20] is a significant feature. This pattern, showing the regional impact of the South Atlantic High (SAH), is well represented both in CONTROL-E40 (Figure 2, second column) and CONTROL-EC4 (Figure 2, third column) runs. Comparison of the original 2.5° × 2.5° ERA-40 with PRECIS outputs shows that the model maintains all the features (both in direction and intensity) present in the original lower-resolution reanalysis. Over the Altiplano region in Bolivia, Chile and northwestern Argentina as well as along the high Andes to the south and north, the model output does have problems since this level is lower than the orography. Furthermore, along the eastern side of these orographic features, CONTROL-E40 flow tends to become perpendicular to them rather than flowing mostly parallel as in the reanalysis. CONTROL-EC4 output also reproduces the wind field's mean behavior, that is, the trade wind deflection by the Andes and the strong flow over Paraguay and Argentina over the southern section of the domain. However, the wind field along the Altiplano and the high Andes also differs from the ERA-40 reanalysis, the former being more northerly while the reanalysis shows a more north-northwesterly flow over Paraguay and northern Argentina and a northeasterly change south of approximately 25°S. CONTROL-EC4 also suffers from the same quasi-perpendicular flow towards high orographic features. CONTROL-EC4 winds along the domain's southern edge also show a different flow (northerly) than the ERA-40 reanalysis and CONTROL-E40 run, the latter better agreeing with Rio de La Plata Basin climatology. Along the Pacific coast, in particular the Peruvian one, CONTROL-EC4 winds tend to be parallel to the coastline at a closer range, differing from the ERA-40 wind field. Arguably such differences could be due to domain edge effects, but these are not observed in CONTROL-E40. The wind intensity over Brazil is somewhat overestimated by CONTROL-E40 run, but the slightly more intense winds along the northern edge of the domain and over the Altiplano differ little from ERA-40. CONTROL-EC4 run shows winds over 3 m/s almost throughout the center of the domain.

During the Austral winter (JJA, Figure 2(c)), the 850 hPa circulation again shows SAH's influence, albeit a somewhat weaker one. Both simulations overall agree with the ERA-40 reanalysis. This time the northwesterly to westerly circulation over southern region of the domain is reasonably well reproduced. Over Bolivia CONTROL-EC4 exhibits slightly weaker winds, as over the domain's northeastern sector. As before, the agreement with AM10 simulations is good and actually closer to ERA-40 than to NCEP. Modeled winds along the Pacific coast again tend to differ from the ERA-40 reanalysis.

The modeled flows during equinoxes (autumn, MAM, Figure 2(b), and spring, SON, Figure 2(d)) are in good agreement with the ERA-40 reanalysis, though somewhat weaker winds tend to occur over the domain's southern third. Other issues noted above also apply to equinoxes.

Comparison with AM10 PRECIS simulation runs over a more extended domain for the period 1961–1990, using NCEP reanalysis and the HadAM3P output as drivers (their Figure 4), shows that current simulation results and reanalysis products are in overall good agreement. ERA-40 reanalysis shows somewhat stronger winds over Bolivia, Paraguay, and northern Argentina than the NCEP one, and both GCM-driven simulations appear to be closer to ERA-40 reanalysis than to the AM10 NCEP one, at least for summer months.

Inspection of ERA-40 and the simulations for the upper tropospheric level show that they are in reasonably good agreement (Figure 3). During summer months (DJF, Figure 3(a)), ERA-40 reanalysis shows the Bolivian High, centered over 16°S, 67°W approximately, the strong southerly flow over central Brazil, associated with summer deep convection, and the Northeast trough [21, 22]. The westerly flow over the southern part of the domain agrees with observations. While the main summer circulation features are reproduced in both simulations, the Bolivian High is only slightly displaced in CONTROL-E40 (Figure 3, second column), but in CONTROL-EC4 (Figure 3, third column) its center is displaced north to 12°S with the resulting large circulation modifications in the domain's northern half, which becomes southerly to southwesterly rather than southeasterly to southerly. Also, the westerly circulation over the southern half of the domain is weaker. Comparison with AM10 (their Figure 5) shows that the Had3AM in PRECIS does a better job of simulating the Bolivian High, in reasonably good agreement with ERA-40 even if the authors noted a bad representation compared to NCEP reanalysis. At higher latitudes their results are also in better agreement with ERA-40 reanalysis.

The winter circulation (Figure 3(c), JJA) is essentially a westerly flow over two-thirds of the domain. CONTROL-E40 run is in good agreement with its source data, with a few minor changes in the jet location over the southern half of the domain. On the other hand, the subtropical jet is not as strong, nor as well defined in CONTROL-EC4, and the flow is stronger and somewhat veered in the domain's tropical sector. AM10 results also have problems simulating the subtropical jet.

During equinoxes, the upper tropospheric wind field, the largest differences between ERA-40 and CONTROL-E40 and CONTROL-EC40 are found in the equatorial section of the domain. The autumn anticyclonic circulation, observed south of the Equator, with center near 6°S, 63°W, is smaller and displaced west and north for CONTROL-E40 and does not appear in CONTROL-EC4. In the latter case the westerly flow in the southern half of the domain is also much weaker. During spring the differences between the source reanalysis and model runs are not as prominent. CONTROL-EC4 shows again the largest differences, with a weaker anticyclonic circulation south of the Equator. Whether this inadequate representation of the upper troposphere spring circulation for CONTROL-EC4 can result in the observed differences in the summer circulation and the Bolivian High is a matter of future research.

### 3.2. Domain Precipitation and Temperature Validation

**Figure 4:**
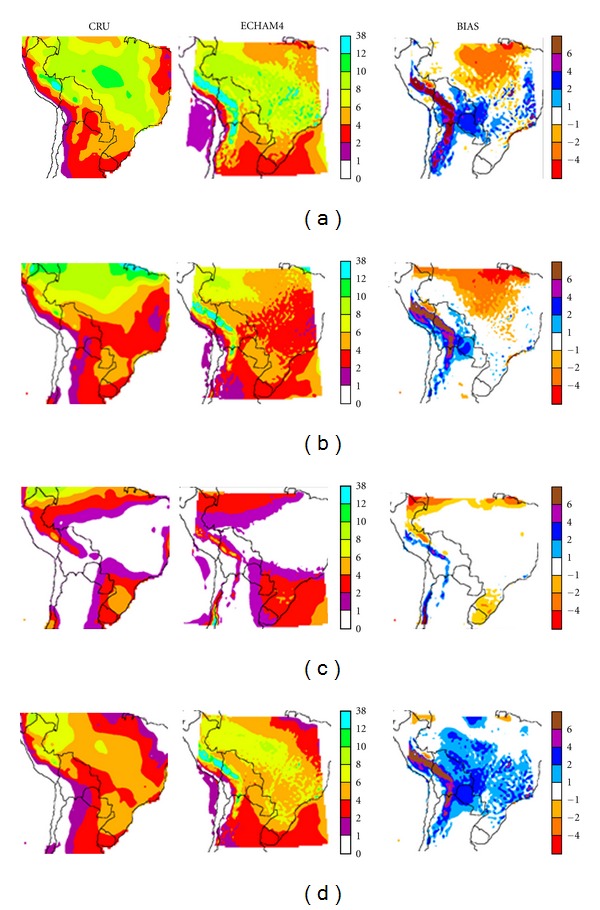
CRU fields (left column) and CONTROL-EC4 (center column) seasonal precipitation, given as precipitation rate (mm day^−1^) for (a) summer (DJF), (b) autumn (MAM), (c) winter (JJA) and (d) spring (SON). The bias, that is, CONTROL-EC4 minus CRU, is shown in the right column.

**Figure 5:**
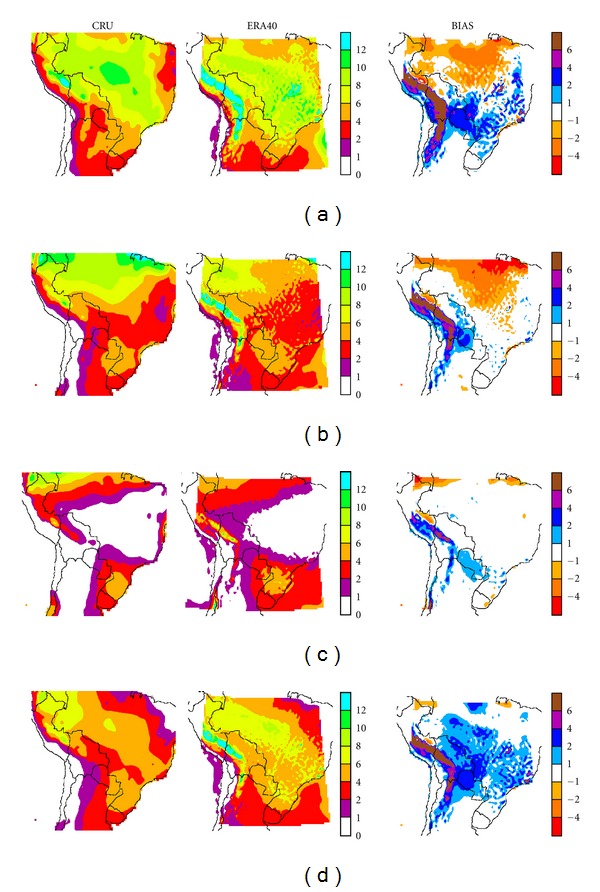
Same as Figure 4, comparing in this case CRU with CONTROL-E40 precipitation rate.

Figure 4 shows the precipitation fields given in mm/day, for CRU precipitation fields, for CONTROL-EC4 run and the model bias with respect to the former. CONTROL-EC4 run, can reasonably reproduce the annual precipitation cycle over the domain, mainly in the tropical regions, with maximum precipitation during the summer (DJF) and a minimum during winter. Similarly Figure 5 shows the same comparisons for CONTROL-E40, which also reproduces the main features of the annual precipitation cycle.

During summer wet season (DJF, Figures 4(a) and 5(a)), the precipitation climatology for model outputs (center) and CRU data (left) shows the main precipitation field feature, a broad high precipitation band on a northwest-southeast axis, extending from Amazonia to southeastern Brazil. Typically in this high precipitation region, simultaneous precipitation events can occur over large areas, for consecutive days [12]. It broadly represents the region's South Atlantic Convergence Zone (SACZ) pattern.

Inspection of the bias plot does show some important differences between the simulated summer precipitation field and observations. CONTROL-EC4 underestimates precipitation over the central region of Amazonia in northern and eastern Brazil, by as much as 6 mm/day, while CONTROL-E40 shows more limited decreases there. Similar differences have been detected in various RCMs/GCMs, such as NASA GISS, CPTEC/COLA, MM5, and HadRM3P (AM10 and references therein), among others. The negative bias in AM10 (their Figure 2) spans an even larger area over Brazil and part of northern Bolivia. Marengo et al. [23, 24] suggested that the radiation parameterization and/or land-surface processes could be associated with such underestimates, and possible effects of local dynamics forcings such as dry or wet soil may be dominant over the large-scale SST forcing. Given that climate, soil, and vegetation interact on many different temporal and spatial scales, it is not simple to reproduce such mechanisms and the resulting errors will remain present both in GCMs and RCMs. Another bias during summer, also present in other GCMs and RCMs is a precipitation overestimate of about 4 mm day^−1^ in Paraguayan and Bolivian Chaco as well as neighboring parts of Brazil. Even larger differences are observed over the Andes, in particular over their eastern slopes, with overestimates up to 6 mm day^−1^. A similar behavior is found for CONTROL-E40 with enhanced precipitation over southern Brazil. Comparison with AM10 shows that the current simulation has a somewhat larger overestimate in the Paraguayan and Bolivian Chaco, but excess precipitation over the Andes does not extend as far south. There is no excess precipitation over Uruguay and Argentina's Mesopotamia (between the Paraná and Uruguay Rivers) and Chaco.

It could be that the inadequate circulation simulation along the Altiplano and the Andes, with a tendency to a more perpendicular flow towards the orographic barriers, together with a more northeasterly flow, also displaced closer to the Andes, can enhance moisture advection there and lead to such precipitation enhancements, in agreement with Da Rocha et al. [12].

During the winter (JJA, Figures 4(c) and 5(c)) dry season, CONTROL-EC4 and CONTROL-E40 runs appear to reproduce all the main features of CRU. Main differences in CONTROL-EC4 arise in southern Brazil and Uruguay, where precipitation is underestimated. Solman et al. [25] obtained a similar result with RCM MM5. Larger precipitation underestimates are also observed along the northwestern edge of the domain in the Peruvian Amazonia and along the northern edge, over Brazil, in agreement with AM10. Similar results, if somewhat less extended, are obtained in CONTROL-E40. As was the case for summer simulations, albeit in more limited areas consistent with the change in circulation and water advection, excess precipitation is observed along the Andes eastern slopes as well as in central Chile along the western slopes. For CONTROL-E40 the enhancement is observed along the Andes western and central slopes in central and southern Peru, consistent with the seasonal circulation. Both CONTROL-E40 and CONTROL-EC4 runs show a stronger flow towards the Andes, in this particular case a westerly flow over central Chile and its Pacific coast. Similar enhanced precipitation areas are also found in ETA model runs [26].

During autumn (MAM, Figures 4(b) and 5(b)), the most extended differences can be observed, for both runs, over the northern half of the domain, where the model underestimates precipitation. This could be due to the seasonal circulation problems noted previously. On the other hand, during spring (SON, Figures 4(d) and 5(d)) the model yields moderately enhanced rainfall with respect to CRU over western Brazil, Paraguay, and Bolivia. During both equinoxes, there is a major precipitation enhancement over the tropical and subtropical Andes.

**Figure 6:**
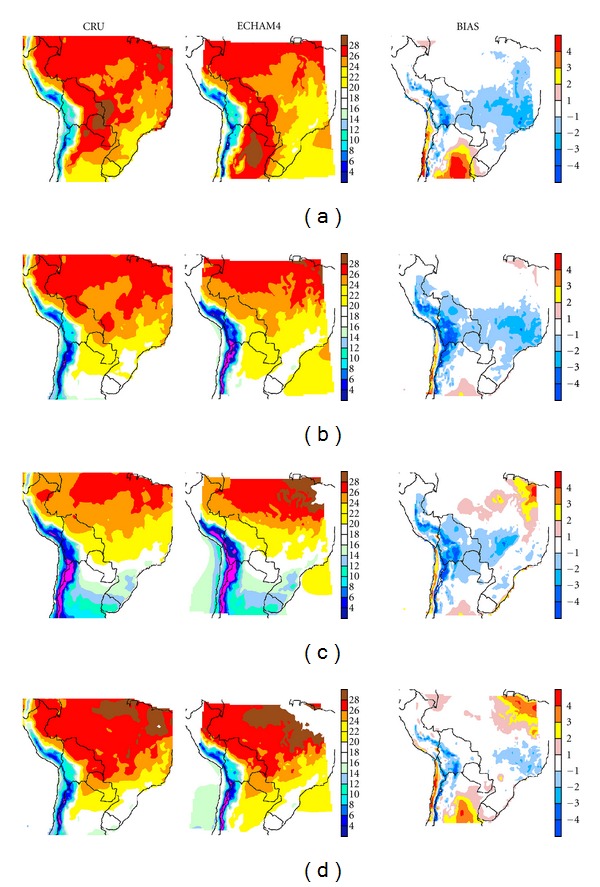
CRU fields (left column) and CONTROL-EC4 (center column) mean seasonal temperature (°C) for (a) summer (DJF), (b) autumn (MAM), (c) winter (JJA) and (d) spring (SON). The bias, that is, CONTROL-EC4 minus CRU, is shown in the right column.

**Figure 7:**
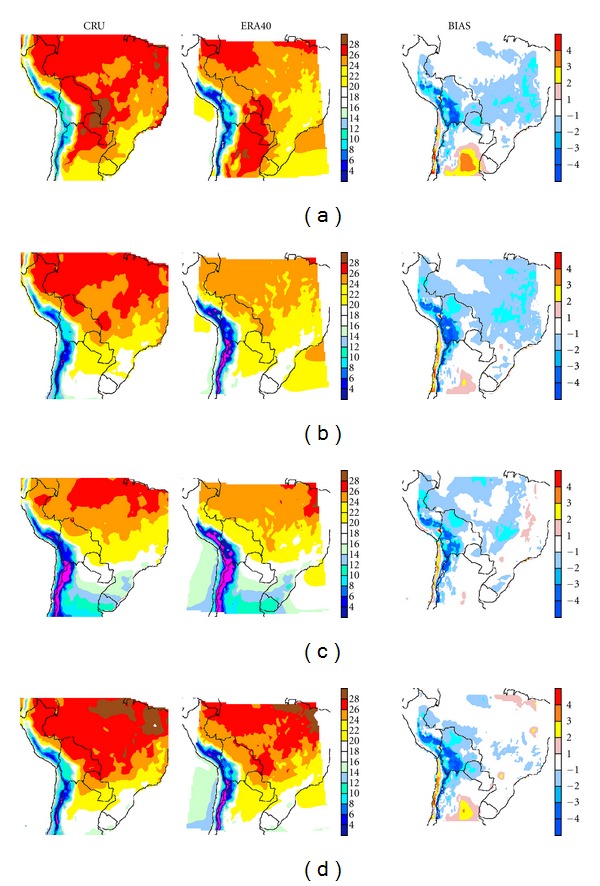
Same as Figure 6, comparing in this case CRU with CONTROL-E40 temperature.

Figures 6 and 7 show the mean seasonal surface temperatures for CONTROL-EC4 and CONTROL-E40 (center), respectively, and CRU data (left). The mean seasonal fields produced by PRECIS reproduce the major temperature features and seasonal cycle observed in CRU. Major summer differences (Figures 6(a) and 7(a)) can be found in the Argentine Chaco region where both runs place the warmest temperatures, whereas CRU shows the warmest temperatures in western Paraguay and over northeastern Brazil. RCM runs appear to yield cooler temperatures over central and southern Brazil, though this feature is more extended in CONTROL-E40. For winter (Figure 6(c)), the domain's northern edge in CONTROL-EC4 appears to be warmer.

Bias estimates (right column) show that both runs, for summer, overestimate temperatures over Argentina's Humid Pampas, Mesopotamia, and parts of Chaco, by as much as 6°C. A positive bias can be also seen over the Pacific desert coast of Peru and Chile, and central Chile. Temperatures are underestimated on the Altiplano by as much as −4°C and in sectors of the Andes, mostly on the eastern slopes. Lower temperatures, mostly between −1°C and −3°C, also systematically appear over central to southeastern Brazil for CONTROL-EC4. For CONTROL-E40 (Figure 7(c)) the negative temperature bias extends into the Peruvian Amazonia as well as central and northeastern Brazil, that is, these anomalies are more widespread.

During winter for CONTROL-EC4 (Figure 6(c)), the cooler region in Brazil is smaller, but over the Andes and the Altiplano the negative bias area is larger. Though milder, the positive bias areas over the domain's south section are still present. A positive, large bias appears on the northeast of the domain over Amazonia, between 1°C and 3°C. For CONTROL-E40 (Figure 7(c)) the negative bias extends along the Andes into the Peruvian Amazonia, northern Bolivia, and parts of Brazilian Amazonia and central Brazil. Positive biases are few and small for the latter run.

During the equinox seasons (MAM, Figures 6(b), 7(b) and SON, 6(d), 7(d), resp.) important regional differences are also present. In particular during autumn, for CONTROL-EC4, an extended cool bias extends from the Andes and Altiplano, the northern Gran Chaco and the Selva Paranaense, with minor warm biases near the Equator and over the Argentine Humid Pampas. For CONTROL-E40 the negative bias practically spans all the Brazilian territory, and only limited warming appears over the Humid Pampas. During spring (Figures 6(d) and 7(d)) the cool bias is mostly confined to the Andes and to the Altiplano in both runs, though for CONTROL-E40 the bias does extend into the low lands of Amazonia and Chaco alongside the mountain range. For both runs sizeable warm biases appear in the Humid Pampas, as well as over northeastern Brazil.

RCMs in general, partly due to the inherent problems in the source AGCMs, have similar problems [23–25]. In this case a particular feature is the strong positive bias in summer, mostly over the Pampas region. AM10 and [23, 25] using PRECIS with HadAM3P did not obtain as large a bias there. Hence, this feature could in principle be due to problems in the ECHAM4 Baseline driver, due to land surface process representation in the AGCM. Yet, because it is also present in ERA-40-driven runs, this suggests that this feature could be at least partially due to the domain considered. In winter AM10 shows a warm bias in the northeast corner of our domain, albeit a weaker one, which however is not present in CONTROL-E40, suggesting that this feature could be related to the AGCMs driving PRECIS. However, comparison with AM10 biases over the domain shows that their run has a cold bias, mainly in summer, over most of Brazil.

### 3.3. Model Response to Land-Use Changes

Both ERA-40- and ECHAM4-driven PRECIS can thus reasonably represent during the 1960–2000 period, in agreement with the previous RCM literature, the main climate features within the chosen domain. Therefore, using both of these drivers, PRECIS is now applied to study the model's response to deforestation/land-use change on the domain as given in mean seasonal precipitation and temperature simulations. The runs span the same period 1960–2000, with the original land-use scenario included in the RCM (CONTROL-E40 and CONTROL-EC4 runs), the 2002 state of land-use [16] labeled MAP1 runs (MAP1-E40 and MAP1-EC4, resp.), and a hypothetical vegetation/land/use map proposed by Nepstad et al. [7] simulating the state of Amazonia in 2030, as shown in Figure 1, labeled MAP2 runs (MAP2-E40 and MAP2-EC4, resp.).

#### 3.3.1. Precipitation

**Figure 8:**
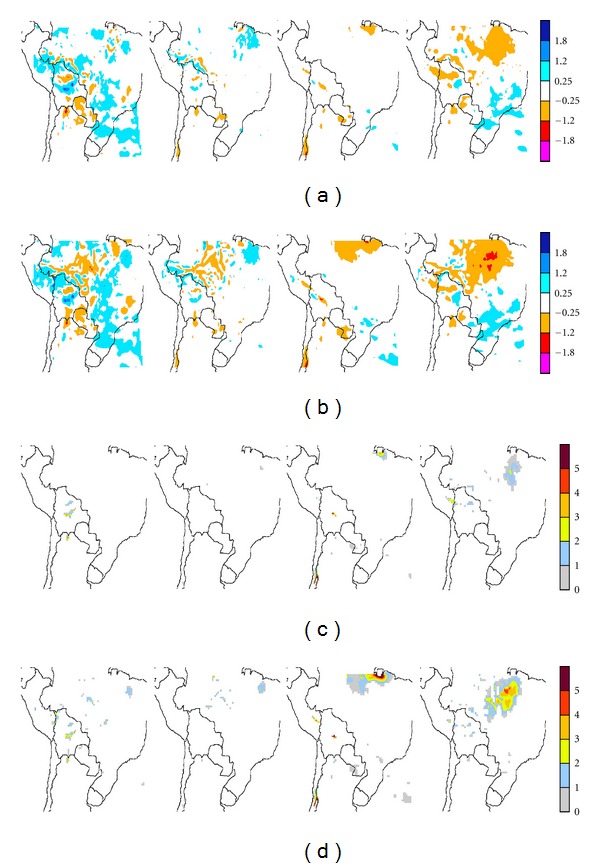
Seasonal precipitation changes, in mm day^−1^, resulting from the two deforestation/land-use scenarios in ECHAM-4 Baseline driven PRECIS runs, for (a) MAP1 and (c) MAP2. The corresponding significances at least at the 90% level are shown in (b) and (d), respectively. Starting from the left the columns correspond to summer (DJF), autumn (MAM), winter (JJA), and spring (SON).

**Figure 9:**
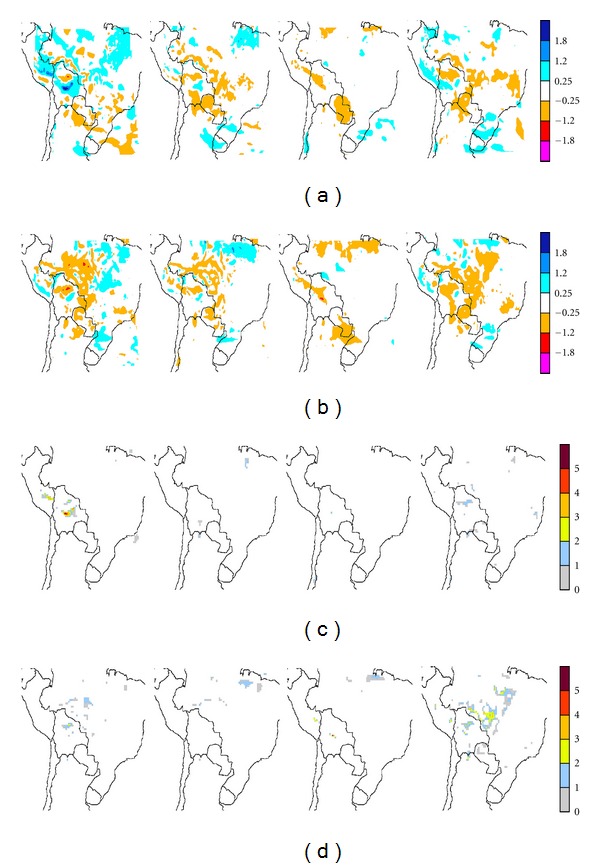
Same as Figure 8 for ERA-40-driven PRECIS runs.

Figures 8 and 9, corresponding to ECHAM4 Baseline and ERA-40, respectively, show the differences in precipitation between CONTROL and the corresponding MAP1 and MAP2 scenarios (rows (a) and (c), resp.), together with the corresponding significance (rows (b) and (d), resp.), for the four seasons (from left to right: summer, DJF, autumn, MAM, winter, JJA, and spring, SON). Statistical significance is given in areas where Student's *t*-test results yield at least 90% significance. A quick overview of rows (a) and (c) shows, for both model drivers, that precipitation is modified mainly in and around areas where land-use change takes place.

Though spatially limited, the larger, more widespread differences with respect to CONTROL are observed in both runs during summer and spring. Both precipitation enhancements and decreases are observed. Note that the differences for MAP1-E40 are less fragmented and usually more extended than for MAP1-EC4. However, areas where the changes are statistically significant are fewer and far more sparsely distributed. The largest discrepancies between the runs appear near the edges of the domain. Inspection of the local statistical significance in precipitation changes shows that statistically significant differences, 90% significant or more, occur primarily in spring and in summer for both ECHAM4 Baseline and ERA-40 runs. The small areas with significant values occur along the edge of the Altiplano region in Bolivia and to a lesser extent in southern Perú. For the ECHAM4 run during spring, and to a lesser extent winter, differences are also significant over northern Brazil, within Amazonia. These changes imply precipitation decreases of about 1.2 mm/day, that is, a 10% decrease along the edge of the Altiplano and a 20% decrease in Amazonia. Note that when these precipitation changes extend beyond deforested areas, such processes occur on the lee side of the latter, according to the 850 hPa circulation (Figure 2). Thus, land-use changes in the tropics could impact on areas not yet affected by such processes. On the other hand, subtropical deforested areas in the Argentine Chaco do not appear to impact upon PRECIS precipitation rates. 

When the 2030 deforestation scenario is considered, both runs yield correspondingly larger impacts (Figures 8 and 9, rows (c) and (d)). In general MAP2-EC4 yields approximately similar areas with decreased precipitation, except in Amazonia, where there appears to be enhancements in all deforested areas, and larger areas beyond with precipitation increases, which however are mostly nonsignificant. Summer precipitation decreases distinctly correspond to the deforested areas, particularly in MAP2-E40, though once more these primarily occur within the western half of the domain. In Amazonia, for both MAP2-EC4 and MAP2-E40, the larger, more widespread decreases appear during winter and spring. During winter the precipitation decreases only span parts of the deforested regions mainly over the Chaco region. In spring, for both runs, these appear in most of the deforested areas particularly in the center of the domain, with larger, statistically significant decreases. In winter and spring areas with significant precipitation decreases extend along the edge of the Altiplano from Perú into Argentina. A winter decrease is also apparent in the border between Paraguay and Argentina. During summer both significant precipitation decreases and a few enhancements are observed in the lee side of deforested areas. Again main precipitation changes occur within the tropics, and the few changes extending into subtropical areas are not statistically significant. 

When the mean domain precipitation is calculated for the three scenarios and the differences estimated (Table 1), it is found that PRÉCIS, even in the case of widespread deforestation corresponding to MAP2 for both ECHAM4 and ERA-40, yields minor regional changes. The minor differences observed for each season are nonsignificant in all cases. In consequence, this implies that on a regional scale PRECIS response does not yield overall significant precipitations changes, even though significant decreases and a few minor enhancements are observed on the local scale within or near deforested areas, even for the worst case 2030 scenario. Furthermore, for both scenarios most of the significant local changes appear within tropical latitudes. 

#### 3.3.2. Temperature

**Figure 10:**
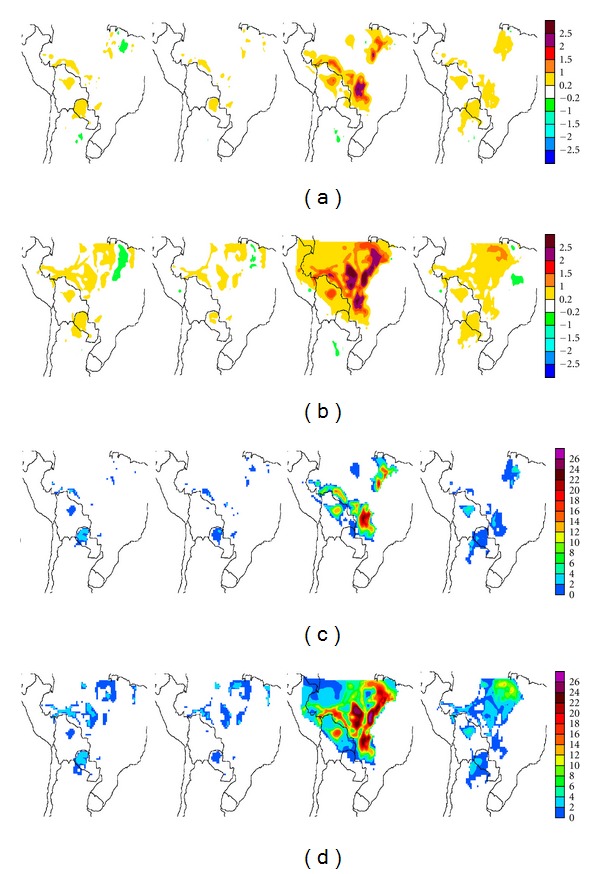
Seasonal temperature changes, in °C, resulting from the two deforestation/land-use scenarios in ECHAM-4 Baseline driven PRECIS runs, for (a) MAP1 and (c) MAP2. The corresponding significances at least at the 95% level are shown in (b) and (d), respectively. Starting from the left the columns correspond to summer (DJF), autumn (MAM), winter (JJA), and spring (SON).

**Figure 11:**
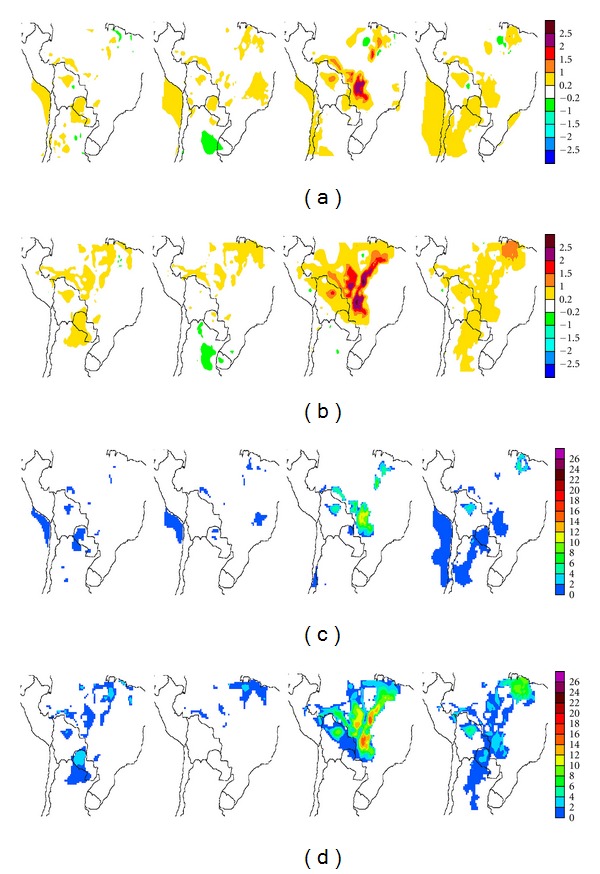
Same as Figure 10 for ERA-40-driven PRECIS runs.

Deforestation/land-use changes result in temperature increases within deforested areas (Figures 10 and 11) for both deforestation scenarios. Maximum temperature enhancements take place during the winter dry season and can be as high as 3°C with respect to CONTROL. In both land-use scenarios more widespread temperature enhancements are observed for ERA-40 than for ECHAM4 Baseline runs. In both cases, only a few weak, spatially limited temperature decreases, always less than 1°C, appear in northeastern Brazil and northern Argentina. However, these are not statistically significant. Statistically significant temperature enhancements beyond deforested areas are observed in MAP1-E40 (Figure 10, rows (a) and (b)) primarily over coastal areas of the Pacific Ocean, off Peruvian and Chilean coasts, in particular during autumn and spring. Some differences are also observed over Argentina, particularly during spring months. While for precipitation changes, few of the differences between scenarios were statistically significant, in the case of temperature almost all the differences within the tropics are. 

From a seasonal perspective, the fewest significant differences with respect to CONTROL arise during autumn. The largest temperature enhancements which arise in winter and spring occur within deforested regions for both scenarios. For MAP1-EC4 (Figure 10, rows (a) and (b)) spring and summer yield only limited, statistically significant changes, at least 95% significant, outside deforested areas in the Argentine and Paraguayan Chaco regions, and in the northeast of the domain. However, during winter, the effects of deforestation/land-use change become significant in large areas well beyond the deforested sectors. For MAP1-E40 (Figure 11, rows (a) and (b)) reduced, spatially limited but significant temperature enhancements extend into Argentina and even Chile during summer and autumn. These become particularly extended during spring months. These enhancements extend beyond Chaco into other ecoregions such as the arid to semiarid provinces of Northwestern Argentina (NOA) and Cuyo, along the Andes' eastern slopes. These enhancements further extend beyond the western slope, the Andes, into the Atacama Desert and central Chile during winter and spring. 

The more extensive deforestation considered in the MAP2 (Figures 10 and 11, rows (c) and (d)) runs yield temperature differences spanning almost all deforested/land-use change areas, particularly within the tropics. During winter extensive areas of warming occur outside these areas for ECHAM4 Baseline and are a widespread occurrence, with statistically significant warming of at least 1°C. Such warming arising in unperturbed areas occur almost throughout the tropical region, mainly to the east of the deforested areas of the domain, spanning Perú, Brazil, Bolivia, and the northern tip of Paraguay. In the MAP2-E40 run (Figure 11, rows (c) and (d)), similar results are obtained during winter months, but the warming areas observed in MAP2-EC4 over coastal areas of the Pacific vanish. During spring a statistically significant warming over Argentina remains, albeit displaced to the east with respect to MAP1 simulations. The winter warming is thus a particularly prominent aspect of the estimated 2030 deforestation scenario (MAP2). Within deforested areas in Brazil, winter temperature enhancements can be as large as 2.5–3°C. 

**Figure 12:**
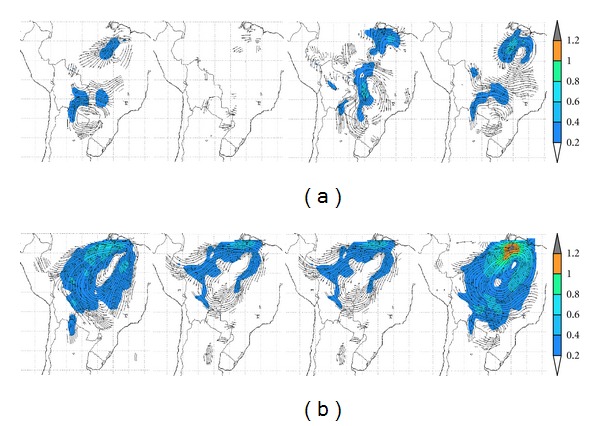
Seasonal 850 hPa wind field differences for ERA-40 driven runs for deforestation/land-use scenarios a) MAP1 and b) MAP2. Starting from the left the columns correspond to summer (DJF), autumn (MAM), winter (JJA) and spring (SON).

The significant warming extending south into Argentina beyond deforested areas, particularly during spring in ERA-40 land-use simulations, needs to be further looked into. These warmings appear in the areas of influence of the Chaco Jet or Low-Level Jet (LLJ) events [21, 22, 27] which are to a large extent responsible, particularly in summer when they most frequently occur, for the meridional water vapor transport into the Pampas region.  Figure 12 shows the ERA-40 seasonal 850 hPa wind field differences with respect to CONTROL-E40 for both deforestation scenarios. 

Inspection of the MAP1-E40 wind difference fields (Figure 12, row (a)) suggests that over the Pacific off the deserts extending along the Chilean and Peruvian coasts, observed temperature enhancements could be linked to these differences. These wind differences can either weaken the southerly flow or enhance the northeasterly flow, over areas where there are statistically significant temperature enhancements. In the Argentine Chaco and Pampas regions wind field differences also suggest an enhanced northerly flow during spring and summer for this deforestation scenario. Furthermore, over deforested regions of the Chaco region in Brazil, near the border with Bolivia and Paraguay, where significant local warming is observed in winter, there appears to be an enhancement of cyclonic circulation, in agreement with such warming. For MAP2-E40 scenario (Figure 12, row (b)) the main feature is the appearance of a strong anticyclonic difference spanning all the deforested areas of Brazil during spring as well as during summer, with a particularly strong anomaly over the northeastern area of the domain, where the largest spring warming is observed, together with a limited precipitation enhancement. Such a difference implies that the circulation is partially enhanced over the north and western portions of Amazonia and weakened over central and eastern Brazil. However, the warming extending into central and central western Argentina in this deforestation scenario does not appear to coincide with detectable wind differences. Such results would suggest that the mechanisms involved in the warming observed well beyond deforested areas do not have a simple explanation in low-level circulation changes that would affect a meridional heat flow. Thus, there may be other competing processes linked to water vapor transport changes that could either enhance or hinder surface warming. The role of the LLJ events in such regional warming processes over Argentina, given their episodic nature, will require further studies beyond the scope of the present study. 

 From a regional perspective, Table 2 shows the statistical significance of the difference between CONTROL and MAP1 and MAP2 average temperature over the domain. Mean regional temperatures in winter for MAP2 scenario, as would be expected, are significantly different from winter CONTROL temperatures, for both ECHAM4 and ERA-40. 

## 4. Discussion and Concluding Remarks 

 Validation of ECHAM4-driven PRECIS for the baseline period shows that the model reproduces the main climate features in the domain, well within the expectations and known limitations of currently available RCMs. Results presented here are in good agreement, in particular, with AM10 results obtained with different PRECIS runs as well as with results from various other models. The comparison with ERA-40, both in its original 2.5° × 2.5° resolution and the ERA-40-driven PRECIS output, also shows PRECIS can reasonably well reproduce the observed state of climate throughout most of the domain. The differences with the CRU dataset are also comparable with other RCM results. RCM precipitation underestimation over Amazonia, observed here, is, for example, a common occurrence in both GCMs and RCMs. 

 A number of studies and campaigns, such as ABRACOS and LBA, have been carried out to observe and model current impacts of deforestation upon precipitation and temperatures in Amazonia (see [28] and references therein, and [1, 12, 15, 29, 30] among others). The observation analysis by Negri et al. [28] for the extensive deforestation in Rondonia, western Brazil, shows that during the dry winter season, specifically August, changes in the diurnal precipitation cycle occur, with enhanced occurrence of cloudiness over the deforested and savanna areas. Satellite data from Special Sensor Microwave Imager showed increases in precipitation over deforested areas under study. However, a long-term analysis did not yield conclusive results regarding widespread impacts of deforestation, due to increases in precipitation in Amazonia since the beginning of large-scale deforestation in the late 1970s. A number of authors have noted that such an increase could be driven by other atmospheric processes, masking the deforestation impacts [31, 32]. Durieux et al. [33], analyzing cloud observations, suggested that while interannual variability does not appear to have changed significantly due to deforestation, land-use change processes modified their seasonal distribution resulting in an enhanced seasonality in cloud cover and hence precipitation. 

Recently, Medvigy et al. [15] modeled the region under study with their variable resolution OLAM climate model, run in mesoscale resolution over Amazonia. They find that, for seasonal mean precipitation, early 1990s and 2050 deforestation/land-use scenarios run under current climate conditions would result in different impacts depending on season. Hence, for winter, with respect to the early 1990s deforestation scenario, they find only minor decreases in Brazil north of the Amazon River, as well as a decrease in Venezuela, Colombia, and Guyana, beyond the boundaries of this study. Total deforestation of the Amazon results in similar but stronger changes in that region. During spring their results show minor decreases over central Amazonia and small increases in the Guyana region and southern Amazonia. Total deforestation results again in similar, but stronger patterns in precipitation changes. Decreases also appear over Bolivia, Paraguay, and Southern Brazil for their 2050 land-use scenario. For total Amazonian deforestation some precipitation increases do appear over Southern Brazil and eastern Paraguay. During summer OLAM runs yields, for the 2050 scenario, both minor increases and enhancements over eastern Amazonia, while, total deforestation results in widespread precipitation decreases there. Such changes also extend over western Amazonia in both scenarios. Decreases and enhancements are also observed over the Bolivian lowlands and Paraguay for the 2050 scenario, while for total deforestation, precipitation decreases occur throughout this part of the domain. Finally during autumn, both deforestation scenarios yield precipitation decrease over central Amazonia and central western Brazil. Enhancements appear over southeastern Amazonia. 

As previously noted overall regional precipitation changes in the PRECIS runs yield mostly nonsignificant precipitation changes, even for MAP2 runs. This agrees with the OLAM 2050 scenario results which, from a regional perspective, are minor in most seasons. When precipitation's spatial distribution is considered, PRECIS precipitation changes over Amazonia during spring are also in agreement with OLAM and to a lesser extent over the rest of the domain. MAP2 summer distribution changes resemble the overall interleafed OLAM precipitation enhancement/decreases. During autumn, the observed changes, albeit more reduced, are again in reasonable agreement with the corresponding OLAM runs. Finally for the dry winter season, the PRECIS run results in similar precipitation decreases close to the Amazon River. Other changes in the rest of the domain are again minor. It is important to note that the lack of significant precipitation changes agrees with Negri et al. [28] who could not observe interannual impacts of deforestation. OLAM runs with total deforestation only found a 2.3% regional decrease in precipitation over the Amazon basin. In consequence, present PRECIS results, without significant regional precipitation changes from partial deforestation scenarios, are in good agreement both with the more sophisticated model and observations. 

Temperature increases within large deforested areas have been documented both locally, for example, [10, 34], and through satellite observations, for example, [28, 35]. Early on, Bastable et al. [34] observed in deforested areas temperature enhancements in the diurnal cycle at the end of the dry season (October), of up to 5°C for midday hours in 10-day averages. Similarly, at the beginning of the wet season, in December, observed differences were of the order of 2°C. Gash and Nobre [10] report, for July at an ABRACOS deforested site, near the western edge of Amazonia, a midday temperature enhancement of about 1°C. With satellite data Negri et al. [28] report over an extended deforested area in the Brazilian state of Rondonia, for August 2000, 2 to 4°C average midday temperature enhancements with respect to the surrounding forests. Observational evidence shows that temperature enhancements occur within the deforested areas while in perturbed savannah areas temperatures do not necessarily change. PRECIS results agree well with all these observations, with largest differences occurring during the winter season. In all cases, it appears to reproduce temperature enhancements within the deforest/land-use areas with a seasonal cycle in agreement with the above results. 

Current PRECIS results show that land-use changes over the Amazon Basin and northern Gran Chaco can have local effects all year round, particularly on temperature. Such changes extend from tropical into subtropical latitudes, into the central region of the Rio de La Plata Basin and the Selva Paranaense/Mata Atlantico. While such local impacts are present, the latter regions however do not appear to suffer significant climate impacts from land-use changes/deforestation processes. In other words land-use changes under current climate conditions, and always according to the model runs carried out so far, result in larger local/regional climate impacts the closer they are to the Equator, in particular within the tropics, in Amazonia and northern Gran Chaco. 

Summing up, PRECIS, driven both by ERA-40 and ECHAM4 is sensitive to land-use changes in agreement with both precipitation and temperature observations and model results. Local/regional impacts are prominent both in Amazonia and northern Gran Chaco ecoregions. Hence, at least in the study of mean precipitation and temperature climatologies, and in particular when analyzing specific processes such as deforestation/land-use changes, it provides a viable modeling platform. Further work is necessary to assess the PRECIS capability to reproduce regional variability, and thus assess the land-use change impacts to precipitation frequency, temperature variability and extreme event occurrences. Additional work is also necessary to assess PRECIS capability to reproduce such a major regional features as Chaco Jet/LLJ events. Furthermore, future runs should include other model drivers, for example, ECHAM5, HadAM3P among others, in order to carry out an ensemble study. 

## Figures and Tables

**Table 1:** Consequences of deforestation/land-use change on the seasonal mean precipitation (mm day^−1^) averaged over the model domain. ΔΦ_CM1_ corresponds to the difference CONTROL map minus MAP1 map. Similarly, ΔΦ_CM2_ is the difference CONTROL map minus MAP2 map. *T-s* is the Student's *t*-test, and *P* > *T* is the probability of occurrence. *S* denotes the significance, if “yes”, the differences are significant, if “no”, then the differences are not significant. Upper half corresponds to ERA40 runs and the lower half to ECHAM4.

	CONTROL	MAP1	MAP2	ΔΦ_CM1_	*T-s*	*P* > *T*	*S*	ΔΦ_CM2_	*T-s*	*P* > *T*	*S*
ERA-40											
DJF	6.14	6.09	6.05	−0.05	0.932	0.3570	Not	−0.09	0.665	0.5082	No
MAM	4.09	4.08	4.07	−0.01	0.185	0.8538	Not	−0.02	0.431	0.6676	No
JJA	1.85	1.82	1.78	−0.03	0.386	0.7011	Not	−0.07	0.947	0.3472	No
SON	4.40	4.39	4.32	−0.01	0.106	0.9156	Not	−0.08	1.334	0.2615	No
ECHAM4											
DJF	5.87	5.84	5.83	0.03	0.5492	0.2922	Not	0.04	0.9227	0.1795	No
MAM	3.47	3.45	3.42	0.02	0.4732	0.3187	Not	0.05	1.0221	0.1552	No
JJA	1.46	1.45	1.44	0.01	0.2814	0.7791	Not	0.02	0.4478	0.3277	No
SON	4.25	4.22	4.19	0.03	0.6010	0.2748	Not	0.06	1.4025	0.0824	No

**Table 2:** Consequences of deforestation/land-use change on the seasonal mean temperature (°C) averaged over the model domain. ΔΦ_CM1_ corresponds to the difference CONTROL map minus MAP1 map. Similarly, ΔΦ_CM2_ is the difference CONTROL map minus MAP2 map. *T-s* is the Student's *t*-test, and *P* > *T* is the probability of occurrence. *S* denotes the significance, if “yes”, the differences are significant, if “no”, then the differences are not significant. Upper half corresponds to ERA40 runs and the lower half to ECHAM4.

	CONTROL	MAP1	MAP2	ΔΦ_CM1_	*T-s*	*P* < *T*	*S*	%Δ_CM1_	*T-s*	*P* < *T*	*S*
ERA-40											
DJF	22.71	22.78	22.79	0.07	−0.497	0.6212	Not	0.08	−0.985	0.3284	No
MAM	20.67	20.85	20.88	0.18	−0.759	0.4526	Not	0.21	−0.123	0.9020	No
JJA	18.48	18.67	18.75	0.19	−1.154	0.2569	Not	0.27	−2.099	0.0401	Yes
SON	21.21	21.33	21.38	0.02	−1.236	0.2141	Not	0.17	−1.735	0.0871	No

ECHAM4											
DJF	23.29	23.30	23.32	0.01	0.1216	0.5482	Not	0.04	−0.131	0.4477	No
MAM	21.40	21.42	21.44	0.02	0.0814	0.5323	Not	0.09	−0.084	0.4665	No
JJA	19.36	19.50	19.73	0.14	−0.239	0.1094	Not	0.72	−3.195	0.001	Yes
SON	22.31	22.37	22.44	0.06	−0.481	0.3158	Not	0.26	−1.003	0.1593	No
